# Constraint-Based Optimized Human Skeleton Extraction from Single-Depth Camera

**DOI:** 10.3390/s19112604

**Published:** 2019-06-07

**Authors:** Ruotong Li, Weixin Si, Michael Weinmann, Reinhard Klein

**Affiliations:** 1 Institute of Computer Science II, University of Bonn, Endenicher Allee 19a, 53115 Bonn, Germany; liruoton@cs.uni-bonn.de (R.L.); mw@cs.uni-bonn.de (M.W.); rk@cs.uni-bonn.de (R.K.); 2 CAS Key Laboratory of Human-Machine Intelligence-Synergy Systems, Shenzhen Institutes of Advanced Technology, 1068 Xueyuan Avenue, Shenzhen University Town, Shenzhen 518055, China

**Keywords:** human skeleton extraction, skeleton constraints, consistency, continuity

## Abstract

As a cutting-edge research topic in computer vision and graphics for decades, human skeleton extraction from single-depth camera remains challenging due to possibly occurring occlusions of different body parts, huge appearance variations, and sensor noise. In this paper, we propose to incorporate human skeleton length conservation and symmetry priors as well as temporal constraints to enhance the consistency and continuity for the estimated skeleton of a moving human body. Given an initial estimation of the skeleton joint positions provided per frame by the Kinect SDK or Nuitrack SDK, which do not follow the aforementioned priors and can prone to errors, our framework improves the accuracy of these pose estimates based on the length and symmetry constraints. In addition, our method is device-independent and can be integrated into skeleton extraction SDKs for refinement, allowing the detection of outliers within the initial joint location estimates and predicting new joint location estimates following the temporal observations. The experimental results demonstrate the effectiveness and robustness of our approach in several cases.

## 1. Introduction

Due to its high relevance for a variety of applications including surveillance, smart environment, gaming, human-robot interaction, or medical rehabilitation, the anticipation of human behavior has gained widespread attentions in recent years. The fundamental prerequisition to this task is the extraction of reliable skeleton regarding human poses that form the basic element within the variety of gestures and can then be analyzed in the context of the respective applications.

However, accurate skeleton extraction is a highly challenging task due to the significant variation encountered in appearance, articulation, as well as the possibly occurring strong occlusions of several body parts and noise in the sensor data. Parameters such as height or bone length exhibit significant variations between individuals and, therefore, cannot be provided in advance but have to be estimated. Especially in marker-less motion capture, this becomes particularly challenging due to the highly dimensional representation of human poses conventionally followed in terms of joint models (joints with their positions and relations to other joints) as well as the large number of underlying degrees of freedom. So far, several approaches have been proposed and successfully applied in commercial entertainment applications such as Microsoft’s Kinect [1] or Intel RealSense D435 etc. Many of these approaches are computationally efficient by relying on an initial body part detection, the accordingly carried out placement of joint location hypotheses, and a final skeleton fitting. Yet, as a result of the large variance in appearance and body poses, occlusions of individual body parts, and sensor noise, the extracted human poses are often inaccurate. In particular, the bone length of the extracted skeletons vary over the time and even skeleton joint can be outside the human body shape. Furthermore, temporal dependence is often not considered.

**Figure 1:**
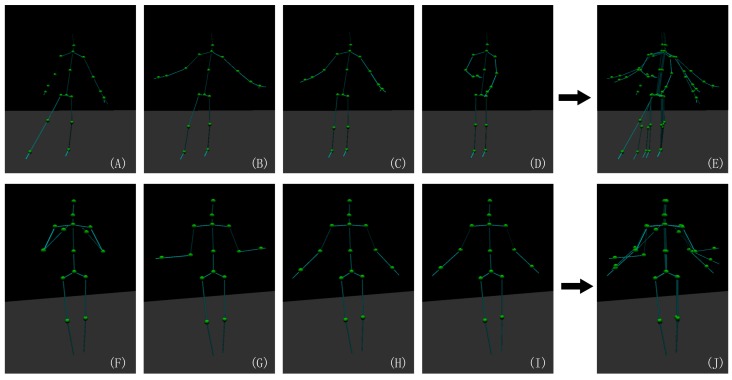
Motion skeleton estimates from two types of motion extraction SDKs. Upper row: The motion skeleton captured by Kinect v2.0. (**A**–**D**) Four frames in the a motion clip and (**E**) all of them in a single image. Bottom row: The motion skeleton captured by Intel RealSense D435 with the Nuitrack SDK. (**F**–**I**) Four frames in a motion clip and (**J**) all of them in a single image. This demonstrates that the respective skeleton estimates vary significantly regarding the bone length during the motion.

**Figure 2:**
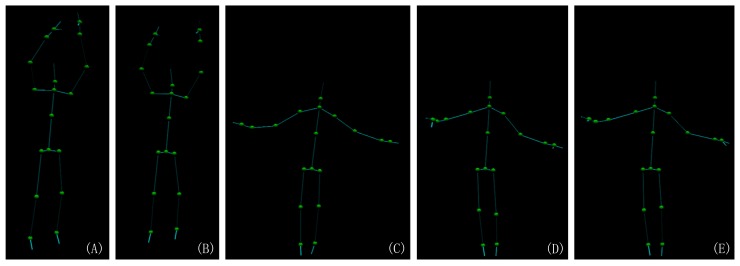
The motion skeleton captured by Kinect v2.0. It shows that the left and right part of the bone are obviously not the same length. In the images (**A**,**B**), the shoulder length and arm length are clearly not symmetrical and (**C**–**E**) the upper-leg and lower-leg are not symmetrical.

**Figure 3:**
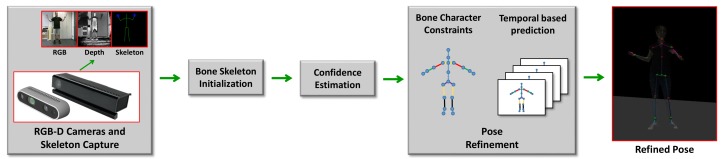
An overview of the proposed pose estimation pipeline given initial possibly corrupted estimations of the skeleton joint positions provided per frame by the pose estimation software for depth cameras, such as the Kinect SDK v2 or the SDK for Intel RealSense D435: Our framework performs a refinement based on symmetry constraints between the left and right body parts as well as the constraint for constant lengths of skeletal parts. Furthermore, outliers detected within the initial joint location estimates are counteracted by additional joint location estimates predicted based on temporal observations.

As shown in Figure 1 and Figure 2, the skeleton captured with the Kinect v2.0 and Nuitrack SDK for Intel RealSense D435 may have an obvious inconsistency regarding the length of the bone section changes over frames and the corresponding left and right body part are not symmetrical. In this paper, we address these challenges by introducing an approach to human pose estimation from a stream of depth images acquired from a single view with additional constraints derived from skeleton characteristics as well as temporal constraints to improve the robustness of of the pose estimates. In particular, we exploit the fact that the lengths of bones remain constant for individual persons within the data and that there are symmetry constraints between left and right body parts. Furthermore, we employ a temporal regularization to consider the continuous nature inherent to human body movements. We integrate these constraints into a nonlinear optimization framework. After an initial per-frame skeleton extraction based on the Kinect SDK [1] or Nuitrack SDK, we determine the reliability of the individual joints in the current frame and apply a refinement that exploits symmetry constraints as well as the constancy of the bone lengths within the skeleton (see Figure 3). Therefore, we also consider the confidence of the skeleton joint estimates based on the orientation of the respective bones to the camera, i.e., if a bone is oriented parallel to the optical axis of the camera, the respective joint observations are likely to be localized more accurately.

The same skeletal constraints as well as temporal characteristics of joint movements are used to detect erroneous estimates by the standard SDKs for depth cameras, as for example, occurring for occlusions of body parts that cannot be compensated by the optimization framework. Erroneous joint location estimates are counteracted by predictions from the observed motion data, i.e., we propagate the reliably extracted information over the temporal domain in order to handle occlusions of body parts possibly occurring in some of the video frames. To counteract the noise in the joint location hypotheses, we use the combination of forward and backward predictions to obtain robust initial joint position estimates, however, at the cost of accepting a delay of three frames. Nevertheless, we favor the accuracy of the estimated skeleton over the true live characteristics. We demonstrate the benefit of this approach on several examples.

In summary, the key contributions of our work include the following:

- We present a novel approach for accurate human skeleton extraction from depth images where the initial per-frame pose estimate delivered by the motion estimation SDKs is refined based on additional symmetry constraints between left and right body parts as well as the constraint for constant lengths of skeletal parts.
- Our framework allows for the detection of errors in the initial joint location estimates and compensates for them based on the joints’ motion characteristics. For this purpose, we use a combination of forward and backward predictions which provide more robust predictions than naive interpolation between data from neighboring frames or simple forward prediction.

Section 2 addresses the previous work of others in pose extraction from RGB-D data. The representation of the human pose and the constraint specifications are explained in Section 3, and the pose refinement steps are detailed in Section 4. In Section 5 and Section 6, we show our results on several cases and discuss the advantages and limitations at the present stage.

## 2. Related Work

Human pose estimation has been extensively studied in literature, and there are several comprehensive surveys on the respective developments [2,3,4].

The most common way for pose representations for human motion analysis is by using skeletons. A skeleton is a tree system of rigid bodies connected by bone joints. Uzun et al. developed a system adapting 3-D models built by an IClone to a human skeleton generated by Kinect [5]. An L1 medial skeleton construction algorithm was proposed to extract skeletons from unoriented raw point clouds [6]. Li et al. [7] proposed a skeleton-based action representation with static and dynamic features. Static features are constructed by the orientation of a rigid body with six rotation matrices, represented by a special orthogonal group. Dynamic features are constructed by the motions of a rigid body represented by a special Euclidean group. They also employ key-skeleton-patterns to find informative skeleton sequences.

Not only does emerging consumer hardware such as Microsoft’s Kinect v2.0 provide the means for low-cost RGB-D measurements but also the various applications in the context of games involve the estimation of human motion. In more detail, the Kinect SDK follows the developments discussed in several publications [8,9,10] and provides a framewise independently performed estimation of human poses from RGB-D data at 30 Hz, even on consumer hardware. Therefore, there is no need for a reliable tracking of the human body over time. Instead, this approach is based on an initial detection of individual body parts based on decision forests that have been trained on synthetic data. The latter has been generated by rendering virtual body models from hundreds of thousands of different poses. In a subsequent step, the locations of different joints are estimated. A comparison regarding the accuracy of joint positions and bone lengths for the first and second generation of the Microsoft Kinect has been conducted by Wang et al. [11]. Han et al. [12] put forward a skeleton-based viewpoint invariant transformation to map 3-D skeleton data to an orthogonal coordinate system built by the left and right shoulders and the spine.

However, the absence of kinematic constraints such as constant bone lengths, collision constraints [13], joint angle constraints [13,14], or velocity and acceleration constraints [15] over the duration of a motion reduces the quality of the resulting pose estimates, in particular for challenging scenarios with complicated poses or occlusions of body parts. Furthermore, the training based on a data set does not generalize to all kinds of users.

Further improvements have been achieved by additionally enforcing kinematic constraints [16], using a multi-channel mixture of parts model with kinematic constraints to improve the robustness of the estimation of the joint positions [17], or encountering the high level of jitter due to noise and estimation errors by applying an extended Kalman filter and the exploitation of sound cues [18]. Furthermore, based on a setup with two Kinects, Yeung et al. [19] approach the human skeleton estimation based on a constrained optimization framework that penalized deviations of the 3-D joint location hypotheses provided by the Kinect SDK for the individual Kinects and that enforced constant bone lengths. Therefore, this technique relies on the quality of the Kinect SDK, and it is impossible to distinguish the front/back directions of the scanned human body. Alternatively, Park et al. [20] used a setup of two cameras from which depth information is calculated and used the RGB-D data to segment human bodies from the background. Then ConVol.utional Pose Machines (CPM) [21] were applied to generate 14 belief maps for each of the considered body parts in the 2-D domain. In a final step, the skeleton was inferred. More recently, Qammaz et al. [22] considered depth information as well as 2-D and 3-D joint hypotheses in their energy optimization framework. Finally, a particle swarm optimization was used.

Furthermore, the refinement of MoCap data has been addressed based on a robust matrix completion approach that takes the low-rank structure and temporal smoothness of motion data into account and is solved using the augmented Lagrange multiplier method [23]. Other approaches [24,25] perform the refinement of human motion data based on dictionary learning approaches which rely on training on high-quality data to allow appropriate results.

Another work directly addresses the inference of human poses solely from RGB information (e.g., [26,27]). The additional use of depth information is expected to result in more accurate pose estimates.

Our approach directly follows the avenue of human pose estimation from RGB-D images captured from a single view. We additionally exploit skeletal constraints such as left/right symmetry besides the bone length constancy as well as temporal motion constraints to refine per-frame pose predictions [8,9,10]. Note that our refinement approach relies on per-frame skeleton estimates as input. While we use initialization based on the Kinect SDK [1], it may also directly be applied to similar input data obtained via different approaches like RealSense SDK or Nuitrack SDK.

## 3. Human Pose Representation and Constraints

Before discussing the steps involved in our pipeline, we provide a discussion of the underlying pose representation and the constraints derived from the skeleton which we use to refine the pose estimates. In the following section, we describe the skeleton pose representation and the proposed constraints.

### 3.1. Pose Representation

**Figure 4:**
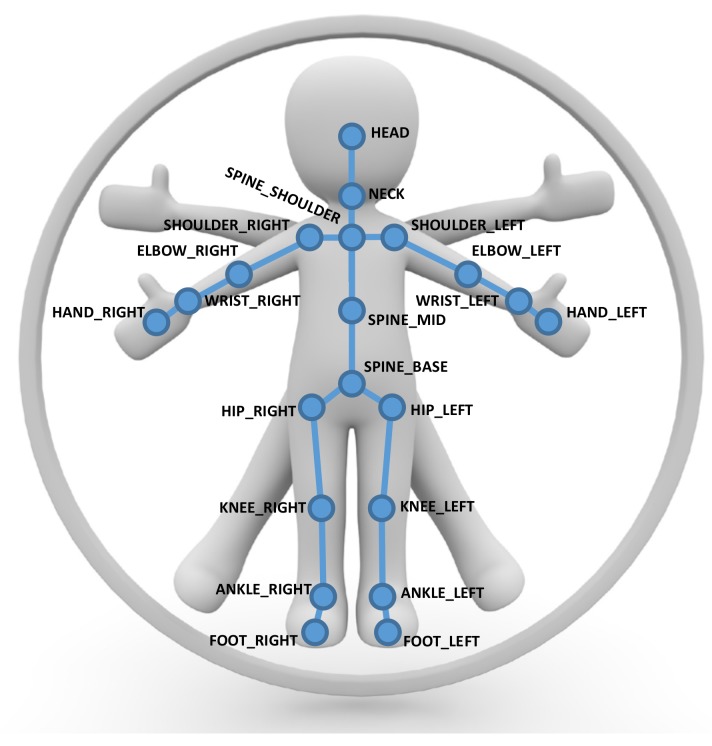
An illustration of the Kinect SDK pose representation using 21 joints with edges corresponding to the bones in between to represent human poses.

**Figure 5:**
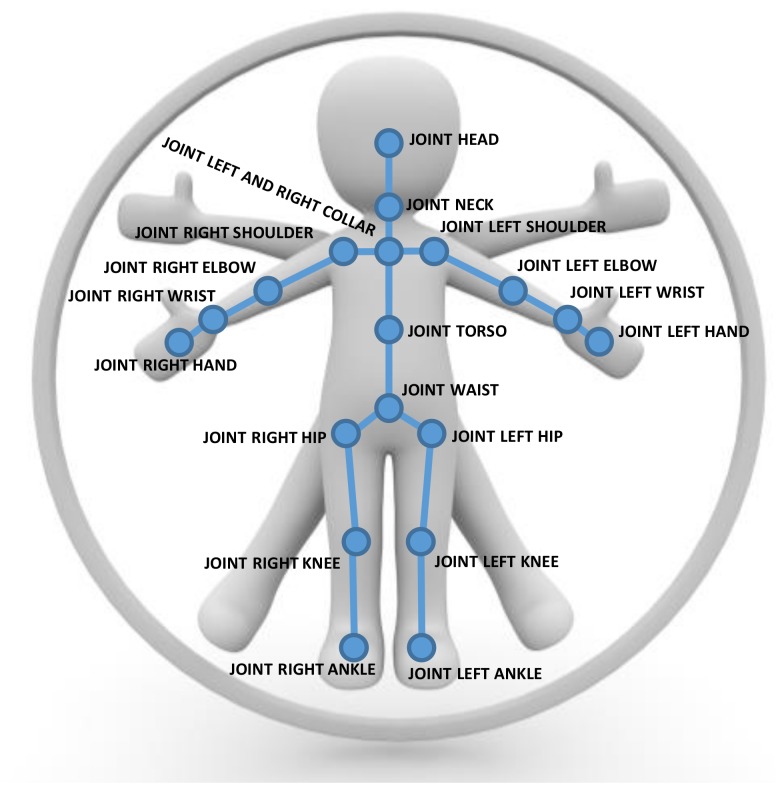
An illustration of the Nuitrack SDK pose representation using 20 joints with edges corresponding to the bones in between to represent human poses.

We represent human poses in terms of a skeletal graph G=(V,E), where vertices $v_{i}\in V$ represent the joints of the skeleton and the edges $e_{ij}\in E$ formed by pairs of certain neighboring joints $\left(v_{i},v_{j}\right)$ correspond to skeletal parts according to the skeleton structure (see Figure 4 and Figure 5). Our skeleton representation is built on the configuration of *N* individual joints, where each joint has certain attached attributes including its position and its tracking state. In comparison to the Kinect SDK [1], we discard the finger tips and the thumbs as they are hard to estimate from typical camera distances with the Kinect SDK and obtain N=21. The skeletons for other SDKs or devices may be defined based on different numbers of joints. These skeleton information allow the inference of the corresponding edge information, i.e., bone information, that contains start and end point and with it the bone length $l_{ij}=|e_{ij}\left|=\right|v_{j}-v_{i}|$ and direction $d_{ij}=v_{j}-v_{i}$ as well as the tracking state.

### 3.2. Skeleton Constraints

**Figure 6:**
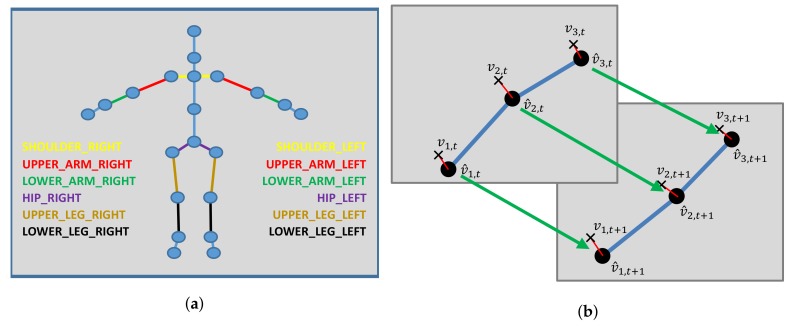
Left: An illustration of the used symmetry constraints. Right: An exemplary illustration of the bone length constancy constraint. (**a**) An illustration of the used symmetry constraints: Shared bone lengths are indicated by the edge colors; (**b**) An exemplary illustration of the bone length constancy constraints: While the joints move from one to the next frame, the bone lengths remain constant.

We exploit constraints that are inherent to the underlying definition of the pose representation in terms of a skeleton. In particular, we exploit the symmetry of the left and right parts of the skeleton including the length of upper and lower arms, the length of upper and lower legs, as well as the distances from the shoulders to the neck that have the same length respectively (see Figure 6a). Furthermore, we exploit the fact that the bone length remains constant over the sequence of the frames (see Figure 6b).

## 4. Robust Human Pose Estimation from RGB-D Data

As illustrated in Figure 3 and Algorithm 1, our approach for robust human pose estimation takes a stream of RGB-D data captured by a depth camera as input, where an initial pose estimate is computed using the Kinect SDK [1] or Nuitrack SDK [28]. In a subsequent refinement step, we exploit the inherent symmetry characteristics of the left and right skeleton parts and the constancy of individual bone lengths over the time and temporal data of body joints to improve the robustness of the resulting pose estimates. In this context, we also introduce a confidence measure for the reliability of the joint estimates derived from the configuration of the bone orientation and the camera orientation. In the following sections, we provide more details regarding the involved steps.

**Algorithm 1** A proposed refinement of initial human pose estimates
**Input:** initial pose estimate with initial joint positions $\left\{j_{i,k}\right\}$ (Section 4.1)
**Output:** refined pose estimate
1:**Bone Lengths Initialization** (Section 4.2): The first *N* frames are used to calculate initial bone lengths based on symmetry and temporal constraints. Bone length hypotheses that strongly deviate for these initial bone lengths are down-weighted. 2:**Trust data detection** (Section 4.3): For new frames, the detected bone lengths are compared to the initial ones. If they deviate significantly or the involved joints are not tracked according to the Kinect SDK, the bone is marked as *not reliably tracked*. 3:**Prediction of joint positions** (Section 4.3): For *not reliably tracked* bones, we predict respective joint positions that are used as initialization to our optimization framework. 4:**Pose refinement based on symmetry and kinematic constraints** (Section 4.4): From the resulting joint positions, we compute a refined pose estimate based on exploiting symmetry constraints and bone length constancy. The optimization is performed using an energy minimization framework.


### 4.1. Initial Per-Frame Pose Extraction

To obtain an initial per-frame pose estimate, we follow the widely used approach implemented in the Kinect SDK [1] that is described in several publications [8,9,10] for Kinect v2.0. This technique relies on the detection of individual body parts based on a random forest based the regression approach and the subsequent estimation of the joint locations. Other softwares like the Nuitrack SDK for the Intel RealSense D435 can also be used to obtain the initial per-frame pose estimation.

However, this per-frame independently conducted pose extraction is lacking regarding the resulting quality as important information inherent to the nature of the skeleton characteristics such as the left/right symmetry of certain body parts or bone length constancy over time as well as the smoothness inherent to human motion are not taken into account. Therefore, there is a need for an incremental refinement of the pose estimates which are the core objective of our work.

### 4.2. Bone Length Initialization

To allow the detection of bad initial per-frame pose estimates, we analyze their bone length characteristics in the first *N* frames of the captured RGB-D input stream. For this purpose, we infer approximate bone lengths for the individual skeleton parts from the distribution of the initial per-frame pose estimates obtained for the first *N* frames.

**Figure 7:**
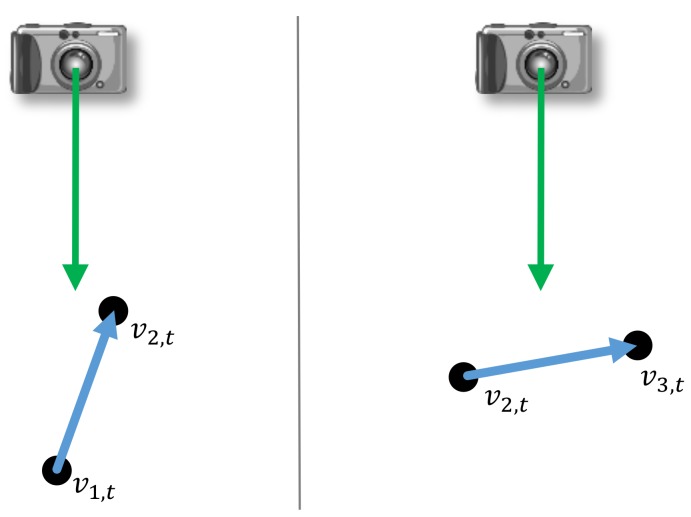
An exemplary 2-D illustration of the confidence of the accuracy of the captured bone lengths. We assume that an individual bone and its respectively connected joints are less accurately determined if the orientations of the bone and the optical axis of the camera are similar (**left**). In contrast, a higher accuracy is expected when the bone orientation is perpendicular to the camera orientation (**right**).

Using the hypotheses $l_{ij,t}$ for bone lengths between the *i*th joint and *j*th joint over the initial *N* frames, we compute the weighted average bone length for the corresponding skeleton part according to

(1) $${\bar{l}}_{ij,\mathrm{ref}}=\frac{\sum_{t=0}^{N}w_{t,\mathrm{angle}}l_{ij,t}}{\sum_{t=0}^{N}w_{t,\mathrm{angle}}},$$

where t=1,…,N. The weights $w_{t}$ represent the confidence of the accuracy of the captured bone lengths which we expect to correlate with the orientations of individual bones and the camera (see Figure 7). In more detail, we assume that an individual bone and its respectively connected joints are more accurately determined when the bone orientation is perpendicular to the camera orientation (i.e., the optical axis of the camera) and less accurately determined if these orientations become more parallel. Therefore, we define the weight $w_{t}$ of an individual bone at time *t* depending on its angle $\theta_{t}$ to the camera as

(2) $$w_{t,\mathrm{angle}}=1-\left|\cos\theta_{t}\right|.$$

In a further step, we analyze the deviations of the individually captured per-frame bone length observations from the reference lengths ${\bar{l}}_{ij,\mathrm{ref}}$ to recalculate the individual bone lengths ${\bar{l}}_{ij,\mathrm{ref}}$, i.e.,

(3) $${\bar{l}}_{ij,\mathrm{ref}}^{*}=\frac{\sum_{t=0}^{N}w_{t}l_{ij,t}}{\sum_{t=0}^{N}w_{t}},$$

where

(4) $$w_{t}=T*\left\{\begin{array}{c} 0,\;\;\;\;\;\frac{d_{ij}}{{\bar{l}}_{ij,\mathrm{ref}}}>0.5\\ 1-\frac{d_{ij}}{{\bar{l}}_{ij,\mathrm{ref}}/0.5},\;\frac{d_{ij}}{{\bar{l}}_{ij,\mathrm{ref}}}\leq 0.5 \end{array}\right.$$

with $d_{ij}=\left|l_{ij}-{\bar{l}}_{ij,\mathrm{ref}}\right|$, and we only allowed 0.5 $l_{ij,ref}$ of the deviation.

(5) $$T=\left\{\begin{array}{c} 1,\;\;\;{\mathrm{state}}_{i}=\mathrm{tracked}\\ 0,\;\;\;{\mathrm{state}}_{i}=\mathrm{notTracked}\\ 0.8,\;\;{\mathrm{state}}_{i}=\mathrm{halfTracked} \end{array}\right.$$

The latter $state_{i}$ takes into account whether a specific bone section is considered as tracked, which is given if the joints on both its sides are tracked. When none of the joints of the respective bone section are tracked, we mark it as *notTracked*. The tracking state information is provided by most of the depth-camera SDKs (for example, the Kinect SKD v2). Furthermore, we use the state of being *halfTracked* when only one of the joints is tracked. The value of *T* is set to 0.8 by experience.

### 4.3. Trust Data Detection and Joint Position Prediction

The joint position estimation provided by the initialization is susceptible to errors due to the huge variations in appearance, possibly occurring occlusions of individual body parts or sensor noise. To detect unreliable initial pose estimates, we analyze the following aspects:

- We analyze the tracking state of the respective bone, i.e., whether it is marked as untracked by the Kinect SDK. If the involved joints are not tracked according to the Kinect SDK, we mark the respective joints as unreliable.
- We analyze whether the deviation of bone lengths is too large. After the initialization phase, we have approximate bone length references that allow for the detection of severely incorrect skeleton pose hypotheses provided by the initial per-frame skeleton estimation. In more detail, we compare the length $l_{ij,t}$ of the bone between the joints *i* and *j* for the current frame *t* obtained from the initial per-frame pose estimates delivered by the Kinect SDK [1] to the corresponding approximate bone length ${\bar{l}}_{ij}$ stored as a reference. If the deviation is larger than a threshold θ (θ=10), we mark the joints *i* and *j* for the current frame as unreliable.
- We analyze whether the joint position is too far away from the predicted position (Section 4.3) to consider the smoothness inherent to the nature of human motion. This means that a joint should only move up to a certain maximum distance between two frames. For this purpose, we analyze the motion characteristics of each joint based on the Mocap Database HDM05 [29] to specify respective thresholds per joint.

**Figure 8:**
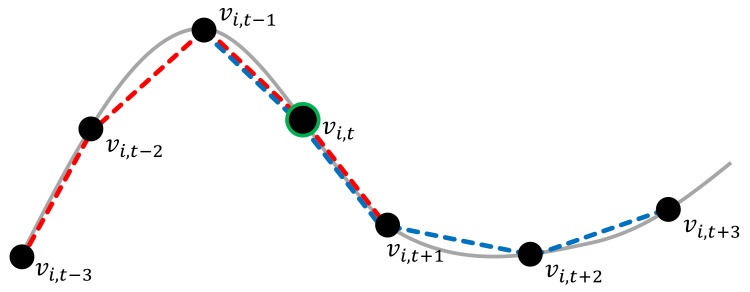
An illustration of the combined forward-backward prediction: To allow a robust estimation that exploits the motion characteristics, we use higher-order approximations of the central differences. Here, we predict the location of $v_{i,t-1}$ based on the previous and the following three frames. The red line connects the joint locations used for forward prediction (Equation (6)) and the blue lines connect the joint locations used for backward prediction (Equation (7)).

As joint locations of $G_{\mathrm{init}}$ may be detected as unreliable, we also predict respective values to counteract unreliable estimates in the subsequent optimization by the algorithm described in Section 4.3. For this purpose, we exploit the smoothness/continuity inherent to human motion, i.e., strong deviations of the positions of a particular joint in adjacent frames are not likely to occur. While a forward prediction from the past frames to the current frame can be efficiently computed, e.g., using finite differences, the noise contained in the joint locations cannot be handled well by such an approach as we observed in initial experiments. To improve the robustness of the prediction, we instead apply a combination of forward and backward predictions (see Figure 8). This means that, in comparison to live pose estimates for the current frame, we perform a trade-off between the live character of the refinement and the accuracy of the initialization that is required to guide the optimizer into the adequate energy minimum. In our case, we accept a delay of three frames (0.1 s for a framerate of 30 Hz) as we consider second-order approximations of central differences assuming constant time steps Δt, i.e., we use a forward prediction according to

(6) $$\begin{array}{c} f_{F}\left(t_{h}\right)=f\left(t_{h-1}\right)+f^{\prime }\left(t_{h-1}\right)\cdot \Delta t\;\;\;\;\\ +f^{\prime \prime }\left(t_{h-1}\right)\cdot \frac{\Delta t^{2}}{2}+O{\left(\Delta t\right)}^{3} \end{array}$$

and a backward prediction according to

(7) $$\begin{array}{c} f_{B}\left(t_{h}\right)=f\left(t_{h+1}\right)+f^{\prime }\left(t_{h+1}\right)\cdot \Delta t\;\;\;\;\;\\ +f^{\prime \prime }\left(t_{h+1}\right)\cdot \frac{\Delta t^{2}}{2}+O{\left(\Delta t\right)}^{3}. \end{array}$$

Here, in Equations (6) and (7), the calculation result of $f_{F}\left(t_{h}\right)$ and $f_{B}\left(t_{h}\right)$ are the predicted location of a joint. If both predictions are nearby, it is an indicator that the initial joint location estimates in the temporally close frames fit together and we take the average as initial position estimate for the respective joint.

(8) $$\bar{f(t_{h})}=\frac{f_{F}\left(t_{h}\right)+f_{B}\left(t_{h}\right)}{2}.$$

If the distance between the forward and backward predicted joint locations are further than a threshold $\theta_{p}$ ($\theta_{p}=8$), the prediction calculated by Equation (8) may not be adequate. We assume that (at least) one of the neighboring frames in either previous or later frame is an outlier. Of course, if the estimates are far from $v_{i}$, it might also be an outlier.

Therefore, computing the estimates for vertices of the sequence will give hints per vertex about its reliability. For each frame, we gave the joint a score $k_{i,t}$, marking the consecutive unreliable. When the non-adequate prediction appears, we mark $k_{i,t}=k_{i,t-1}+1$, otherwise $k_{i,t}=0$.

### 4.4. Pose Refinement Based on Skeleton Constraints

We formulate the refinement of the initial pose estimation $G_{\mathrm{init}}$ given joint position constraints and bone characteristic constraints as an energy minimization problem:

(9) $${arg min}_{{\hat{G}}_{t}}\lambda_{1}E_{1}+\lambda_{2}E_{2},$$

where the first term

(10) $$E_{1}=\sum_{t=1}^{f}\sum_{i=1}^{n}(w_{i,t}∥v_{i,t}-{\hat{v}}_{i,t}{∥}^{2}+u_{i,t}∥p_{i,t}-{\hat{v}}_{i,t}{∥}^{2})$$

penalizes deviations of the optimized joint positions ${\hat{v}}_{i,t}$ of the skeleton graph ${\hat{G}}_{t}$ from the initial per-frame skeleton joints $v_{i,t}$ and the reference skeleton joints $p_{i,t}$ that have been obtained via the aforementioned prediction (Equation (8)). The second term

(11) $$E_{2}=\sum_{t=1}^{f}\sum_{i=1}^{n}{∥l_{ij,t}-{\hat{l}}_{ij,t^{*}}∥}^{2}$$

enforces the bone length constraints based on the exploration of symmetry and bone-length constancy over the temporal domain denoted by the index *t*. We apply a quasi-Newton solver for this energy minimization problem.

The weight $u_{i,t}$ takes the number $k_{i,t}$ of occurring unreliable location estimates of a particular joint *i* in consecutive frames into account. As explained in Section 4.3, a large value of $k_{i,t}$ does not necessarily mean the location of the joint is failed because all of its neighbors might be outliers. However, when the value of $k_{i,t}$ of a vertex in a frame is higher than its value in both neighbor frames, it is surely an outlier. For $k_{i,t}$ consecutive unreliable estimates, the weight is given by

(12) $$u_{i,t}=\exp\left(-\frac{k_{i,t}^{2}}{2\sigma^{2}}\right),$$

where we use σ=3. By specifying

(13) $$w_{i,t}=1-u_{i,t},$$

thereby we balance the influence of the prediction and the initial observations.

## 5. Experiments and Result

In the scope of the evaluation, we analyze the effect of our refinement on the initial estimates provided by the Kinect SDK and the stability of the resulting bone lengths. Furthermore, we provide a discussion of the effects of our trust detection and motion prediction steps.

The evaluation is performed based on RGB-D video inputs provided by a Kinect v2.0, where the initial skeleton and the respective point cloud are provided by the Kinect SDK [1]. As demonstrated, our skeleton refinement approach results in an improved accuracy and a more robust estimation of the skeleton in comparison to the skeleton provided by the Kinect SDK [1].

### 5.1. Skeleton Correction

**Figure 9:**
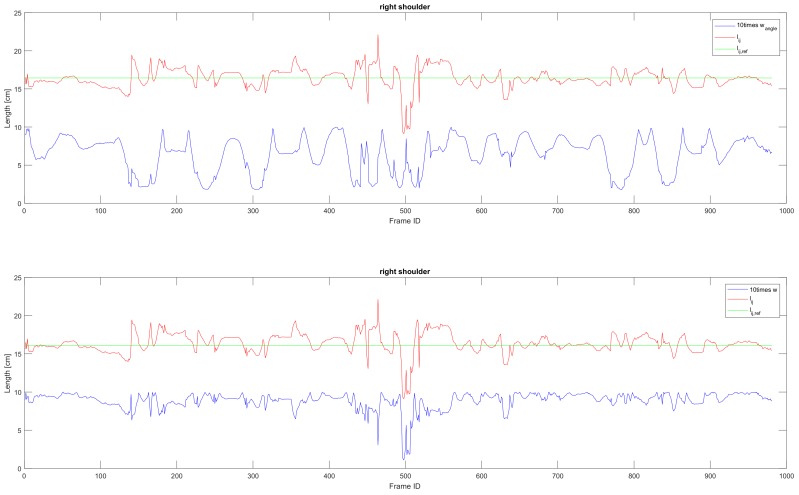
Upper: The relation between $w_{angle}$ and bone length. Lower: The relation between *w* and the bone length. Red line: Bone length observed by Kinect SDK [1]. Green lines: The reference length of a bone section calculated via Equation (3). Blue: Ten times the value of weight $w_{t,angle}$ and $w_{t}$ calculated respectively from Equations (2) and (4).

The skeleton captured by Kinect SDK [1] is very unstable. Especially the length of each bone section in the motion skeleton is changing heavily during the movement (Figure 9) as the relevant direction between bone and camera (illustrated in Figure 7) is changing during the movement of the human. We calculate the reference bone length with Equation (3) in which the weight of the bone length is higher when the bone length is more stable. Figure 9 shows an example of how the length of bone section and its corresponding weight $w_{t,angle}$ and $w_{t}$ change during time while the relevant direction between bone and camera (shown in Figure 7) is changed.

**Figure 10:**
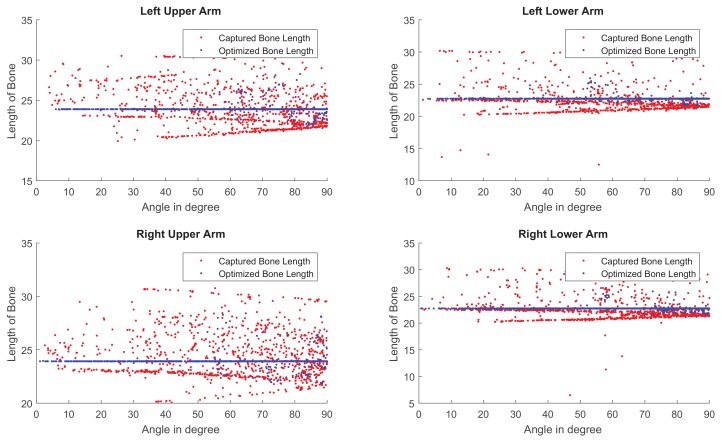
The bone length estimation of four different sections in the skeleton of a motion clip shown respectively in four diagrams. Red: The bone length estimated from the skeleton observed by Kinect SDK [1]. Blue: The bone length estimated via our method.

As shown in Figure 10, the length of the bone is more stable when applying our algorithm on the captured data. The skeleton extracted by the Kinect SDK [1] is more accurate when the bone is parallel to the camera plane, and the length has a larger variance when it is perpendicular to the camera plane. The reason for this deviation is caused by the inaccuracy of the depth camera and the self occlusion of the actor. Therefore, our algorithm downweights the inaccurate bones among the frames (Figure 9). In our algorithm, we implement a reference bone length. The first calculation with Equation (1) aims to remove the influence on the statelessness caused by the direction changing during the movement. And the final reference length for the optimization step is calculated with Equation (3).

### 5.2. Bone Length Derivation

**Figure 11:**
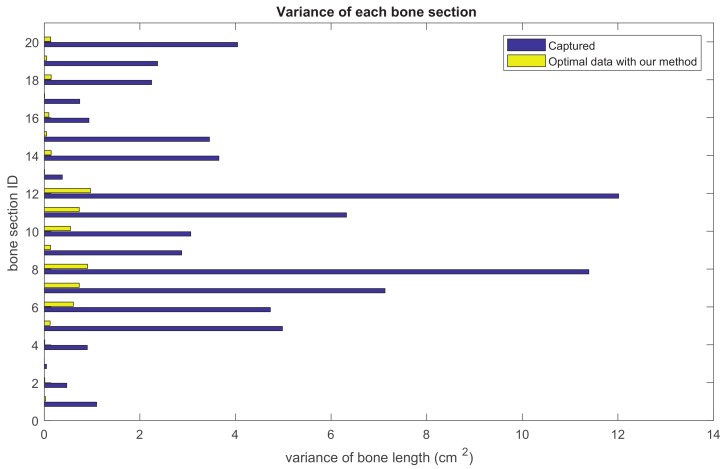
The summation of the bone length variance for each bone section over all frames. Purple: variance of the bone length captured with Kinect SDK [1]. Yellow: variance of the optimized bone length after applying our method.

**Figure 12:**
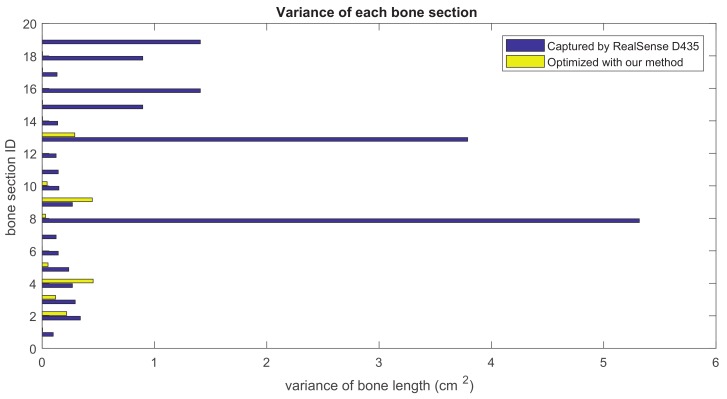
The summation of the bone length variance for each bone section over all frames. Purple: variance of the bone length captured with Nuitrack SDK for RealSense D435. Yellow: variance of the optimized bone length after applying our method.

**Figure 13:**
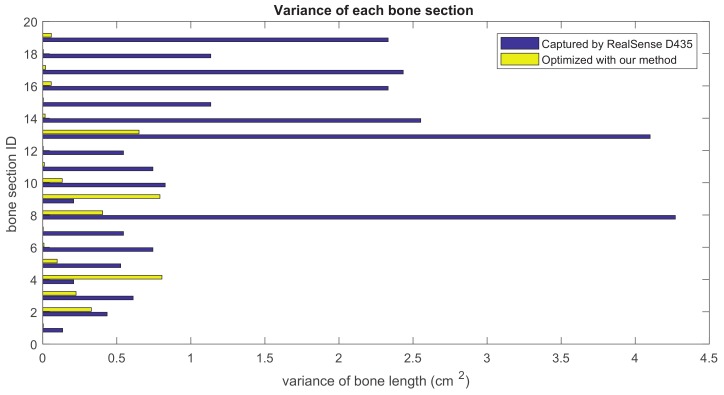
The summation of the bone length variance for each bone section over all frames. Purple: variance of the bone length captured with Nuitrack SDK for RealSense D435. Yellow: variance of the optimized bone length after applying our method.

**Figure 14:**
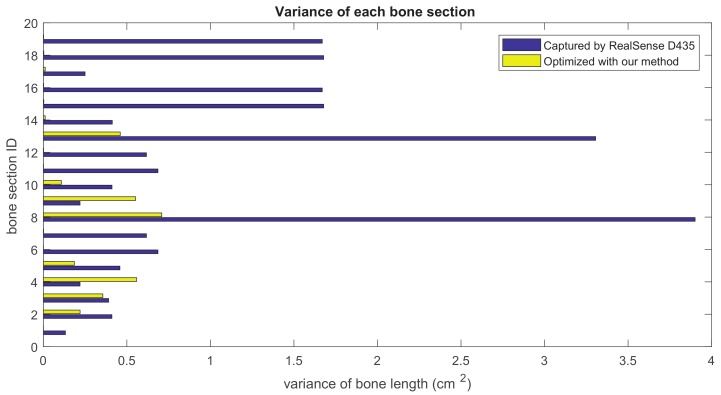
The summation of the bone length variance for each bone section over all frames. Purple: variance of the bone length captured with Nuitrack SDK for RealSense D435. Yellow: variance of the optimized bone length after applying our method.

**Figure 15:**
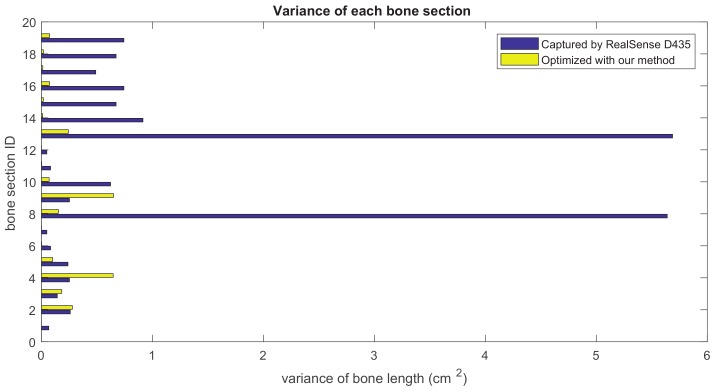
The summation of the bone length variance for each bone section over all frames. Purple: variance of the bone length captured with Nuitrack SDK for RealSense D435. Yellow: variance of the optimized bone length after applying our method.

Our method formulates the problem of skeleton pose estimation as a constrained energy minimization problem. During the update over time, the value of the energy function in Equation (9) decreases. Figure 11 shows the variance of bone lengths of the skeleton captured with the Kinect SDK [1] and the bone variance before and after our optimization steps. Figure 12, Figure 13, Figure 14 and Figure 15 clearly demonstrate that the variance of the bone length in the skeleton estimation by the SDK is obviously reduced after applying our refinement. It also showed that the variance of the bone length is decreased after applying the energy minimization method with bone characteristic constraints.

### 5.3. Trust Detection and Motion Prediction

When human movement is occluded by objects or the self, the Kinect SDK [1] may detect non-relevant objects as parts of the body and this leads to temporal gaps between reliably estimated skeletons. We check the physical characteristics of the joint movement for the detection of erroneous estimates and calculate the physical prediction of the joint position to overcome the errors. In this analysis, we calculate the joint position with a combination of forward and backward predictions (detailed in Equation (8)).

**Figure 16:**
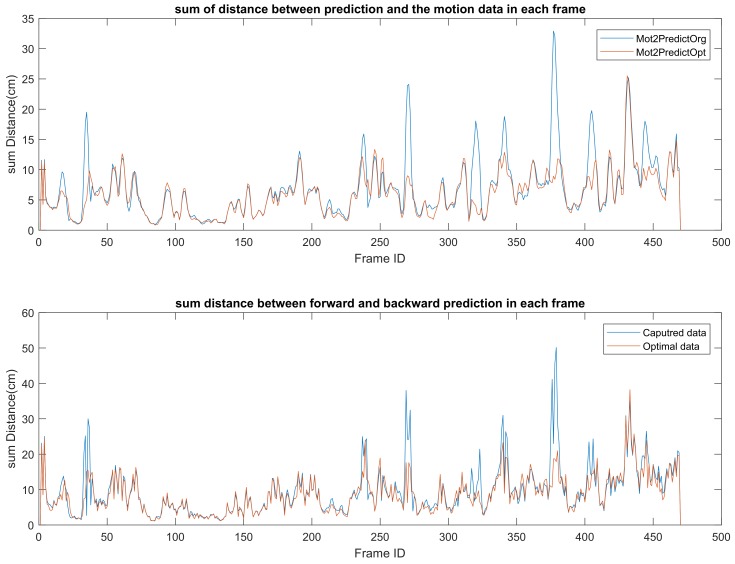
Upper diagram: Summation of the distance between the forward and backward predicted joint positions in each frame. It shows how far the distance is between the estimated joint and the physical predicted position. Bottom diagram: Summation of the distance between joint position and predicted joint position in each frame. It illustrates the temporal symmetry of the motion. Blue: original captured data; Red: motion after optimization.

The upper diagram in Figure 16 shows the distance between the predicted position of a skeleton joint and the joint position captured by the Kinect SDK [1]. It clearly shows that, after applying our optimization method, the difference between the Kinect SDK [1] extracted joint position and the physical predicted joint position is decreased.

Furthermore, when we evaluate a “good” motion, one of the standers is temporal symmetry. This means that the joint trajectory in a smooth motion should be predictable from both the previous and future side. For each time frame, the prediction of the joint position from the previous frame and the future frame has huge a difference in the skeleton captured by the Kinect SDK [1], while when using our method, the difference has decreased (shown in the lower diagram in Figure 16).

### 5.4. Specification of Weights $\lambda_{1}$ and $\lambda_{2}$

In order to find the best value for $\lambda_{1}$ and $\lambda_{2}$, we calculated the value of both energy and variance for different combinations. As a result of a grid search, we obtained $\lambda_{1}=0.2$ and $\lambda_{2}=8.25$ as suitable parameter choices.

### 5.5. Evaluation of Visual Quality

**Figure 17:**
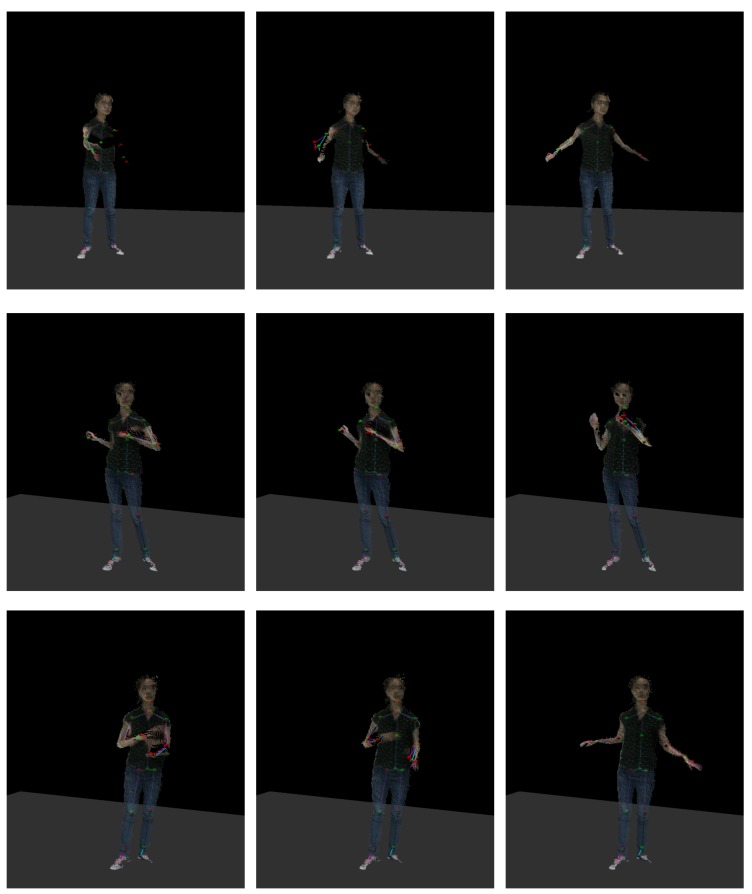
Each row shows three frames in a motion clip. The initial skeleton observed by the Kinect SDK [1] is colored in magenta, and the cyan one is the updated result from our method.

Figure 17 shows screenshot results obtained with our method. With the reference of the point cloud provided by the Kinect SDK [1], it becomes apparent that the updated skeletons are is more stable and accurate than the original captured ones. In some cases, such as in the second frame of the first row and third row, the motion is smoother and more accurate after we apply the prediction.

**Figure 18:**
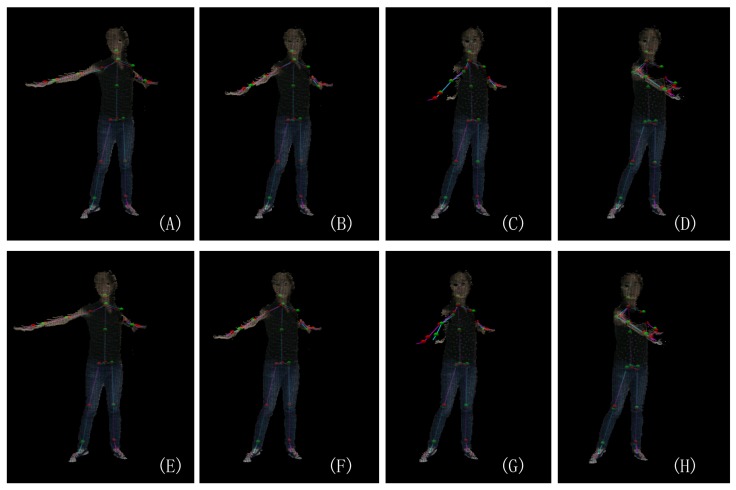
The same four frames of a motion clip are shown: (**A**–**D**) A comparison between the initial skeleton observed by the Kinect SDK [1] (Magenta) and the updated result with forward prediction (Cyan) and (**E**–**H**) a comparison between the initial skeleton observed by the Kinect SDK [1] (Magenta) the updated result with both forward and backward prediction (Cyan).

Especially in the case when the moving speed of a joint is changing, our method with forward and backward predictions allows for the correction of erroneous estimates. Figure 18 shows such an example.

## 6. Discussion

The bone length constraint and the skeleton symmetry constraint together preserve the consistent skeleton along the sequence. When we consider body joints as moving points in the space, their movement should always follow the physical law of kinematics. Therefore, we address the method of trust data detection to check the erroneous cases (Section 4.3). Thus, this method can detect the non-tracked and incorrectly tracked skeleton joints in a single frame or a sequence of frames.

As a result, the length of the skeleton bones are stable and consistent. In addition, our framework not only allows for the detection of outliers within the initial joint location estimates, but also, in such cases, allows for the prediction of new joint location estimates following the temporal observations. As shown in Figure 18, even the hand joints in a motion sequence are estimates as “tracked” from the SDKs, one of the estimated hand joints is actually located outside of the point cloud. Our method involves the temporal information; uses the physical prediction penalizes the initial joint positions, which are far away from the predicted locations (using Equation (10)); and “drags” the joint positions to more precise locations.

**Figure 19:**
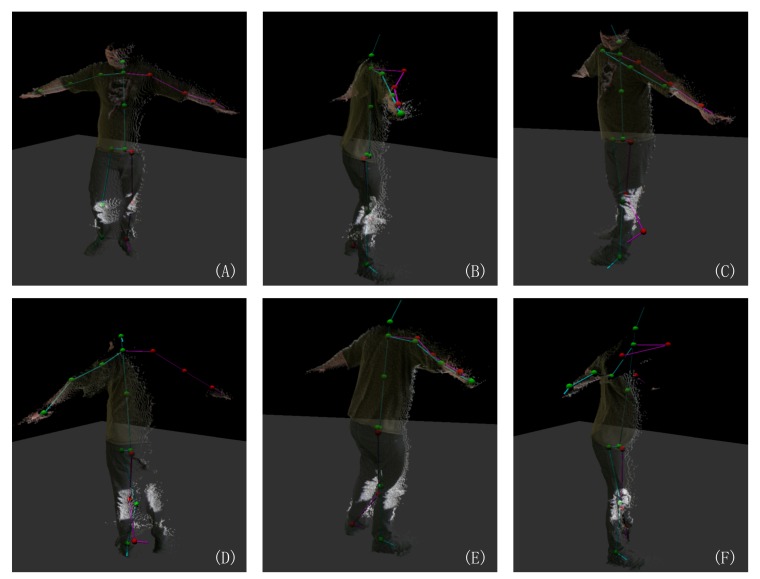
The skeleton observed by Kinect SDK [1] colored in pink and green indicates the left and right side of the body skeleton. It shows that the skeleton observed by the Kinect SDK [1] sometimes (partially) flips when the actor performs a turn-around movement. (**A**) A normal skeleton: pink indicates left, and green indicates right. (**D**) A flipped frame where the right skeleton part is mapped to the left body part and vice versa. (**B**,**C**,**E**) Partial flipped frames in which the skeleton of the left and right arms are mapped in the same direction. (**F**) A failure case.

However, our optimization scheme relies on the absence of longer temporal sequences of unreliable estimates in the original captured data. A special case is turning around, the optimization does not work as there is a left-right flip in the captured skeleton (examples shown in Figure 19). Also, using finite differences to predict the joint position leads a three frames delay of the optimization.

## 7. Conclusions

This paper presented an approach for optimizing human skeleton data acquired by a single depth camera. The skeleton refinement is based on an energy optimization framework that incorporates skeleton characteristics (such as stabilization and symmetry characteristics) as well as the temporal constraints which represent the physical movement of body. In addition, the proposed refinement can be applied to different skeleton representations used in different frameworks/SDKs.

Our method can also be employed to detect erroneous estimates in motion skeleton sequences. Current limitation are that our technique only supports a recovery from errors for up to four erroneous frames in a pose sequence. As modern depth cameras usually have a high frame rates (for example, 30 fps of Kinect v2.0 and 90 fps of Intel RealSense D435), a self-collision motion like turning around may easily take more than 4 frames in the motion sequence. Figure 19 shows a typical failure case of our technique. As our current method can detect these failure cases, we can continue to detect the (partial) flip of a motion skeleton in future work and refine the flipped skeleton. To tackle the above limitations, we plan to couple the convolutional neural network (CNN) couple with long short-term memory networks (LSTM) for continuous pose estimation refinement.

## References

[1] Microsoft (2014). Kinect for Windows. https://developer.microsoft.com/en-us/windows/kinect.

[2] Moeslund T.B., Hilton A., Krüger V. (2006). A Survey of Advances in Vision-based Human Motion Capture and Analysis. Comput. Vis. Image Underst..

[3] Poppe R. (2007). Vision-based Human Motion Analysis: An Overview. Comput. Vis. Image Underst..

[4] Chen L., Wei H., Ferryman J. (2013). A Survey of Human Motion Analysis Using Depth Imagery. Pattern Recognit. Lett..

[5] Uzun Y., Bilban M., Arıkan H. Creating A 3D Model on A Human Skeleton Using Kinect. Proceedings of the 3rd International Conference on Engineering and Natural Sciences (ICENS 2017).

[6] Huang H., Wu S., Cohen-Or D., Gong M., Zhang H., Li G., Chen B. (2013). L1-medial skeleton of point cloud. ACM Trans. Graph..

[7] Li G., Liu K., Ding W., Cheng F., Chen B. (2018). Key-Skeleton-Pattern Mining on 3D Skeletons Represented by Lie Group for Action Recognition. Math. Probl. Eng..

[8] Shotton J., Fitzgibbon A., Cook M., Sharp T., Finocchio M., Moore R., Kipman A., Blake A. Real-time Human Pose Recognition in Parts from Single Depth Images. Proceedings of the 2011 IEEE Conference on Computer Vision and Pattern Recognition, CVPR ’11.

[9] Shotton J., Girshick R., Fitzgibbon A., Sharp T., Cook M., Finocchio M., Moore R., Kohli P., Criminisi A., Kipman A. (2013). Efficient Human Pose Estimation from Single Depth Images. IEEE Trans. Pattern Anal. Mach. Intell..

[10] Kohli P., Shotton J. (2013). Key Developments in Human Pose Estimation for Kinect. Consumer Depth Cameras for Computer Vision.

[11] Wang Q., Kurillo G., Ofli F., Bajcsy R. Evaluation of Pose Tracking Accuracy in the First and Second Generations of Microsoft Kinect. Proceedings of the 2015 International Conference on Healthcare Informatics.

[12] Han Y., Chung S.L., Yeh J.S., Chen Q.J. (2014). Skeleton-based viewpoint invariant transformation for motion analysis. J. Electron. Imaging.

[13] Sminchisescu C., Triggs B. (2003). Estimating Articulated Human Motion with Covariance Scaled Sampling. Int. J. Robot. Res..

[14] Deutscher J., Reid I. (2005). Articulated Body Motion Capture by Stochastic Search. Int. J. Comput. Vis..

[15] Yamamoto M., Yagishita K. Scene constraints-aided tracking of human body. Proceedings of the IEEE Conference on Computer Vision and Pattern Recognition.

[16] Calderita L.V., Bandera J.P., Bustos P., Skiadopoulos A. (2013). Model-Based Reinforcement of Kinect Depth Data for Human Motion Capture Applications. Sensors.

[17] Martínez Berti E., Sánchez Salmerón A., Ricolfe Viala C., Nina O., Shah M. (2016). Human Pose Estimation for RGBD Imagery with Multi-Channel Mixture of Parts and Kinematic Constraints. WSEAS Trans. Comput..

[18] Shu J., Hamano F., Angus J. (2014). Application of extended Kalman filter for improving the accuracy and smoothness of Kinect skeleton-joint estimates. J. Eng. Math..

[19] Yeung K., Kwok T., Wang C. (2013). Improved Skeleton Tracking by Duplex Kinects: A Practical Approach for Real-Time Applications. ASME. J. Comput. Inf. Sci. Eng..

[20] Park S., Ji M., Chun J. (2018). 2D Human Pose Estimation based on Object Detection using RGB-D information. KSII Trans. Internet Inf. Syst..

[21] Ramakrishna V., Munoz D., Hebert M., Andrew Bagnell J., Sheikh Y. (2014). Pose Machines: Articulated Pose Estimation via Inference Machines. Proceedings of the 13th European Conference on Computer Vision—ECCV 2014.

[22] Qammaz A., Michel D., Argyros A.A. A Hybrid Method for 3D Pose Estimation of Personalized Human Body Models. Proceedings of the IEEE Winter Conference on Applications of Computer Vision (WACV 2018).

[23] Feng Y., Xiao J., Zhuang Y., Yang X., Zhang J.J., Song R. (2014). Exploiting temporal stability and low-rank structure for motion capture data refinement. Inf. Sci..

[24] Wang Z., Feng Y., Liu S., Xiao J., Yang X., Zhang J.J. A 3D Human Motion Refinement Method Based on Sparse Motion Bases Selection. Proceedings of the 29th International Conference on Computer Animation and Social Agents, CASA ’16.

[25] Wang Z., Liu S., Qian R., Jiang T., Yang X., Zhang J.J. Human motion data refinement unitizing structural sparsity and spatial-temporal information. Proceedings of the IEEE International Conference on Signal Processing (ICSP).

[26] Yasin H., Iqbal U., Krüger B., Weber A., Gall J. (2015). 3D Pose Estimation from a Single Monocular Image.

[27] Iqbal U., Doering A., Yasin H., Krüger B., Weber A., Gall J. (2018). A dual-source approach for 3D human pose estimation from single images. Comput. Vis. Image Underst..

[28] (2019). NUITRACK SDK. https://nuitrack.com/.

[29] Müller M., Baak A., Seidel H.P. Efficient and Robust Annotation of Motion Capture Data. Proceedings of the ACM SIGGRAPH/Eurographics Symposium on Computer Animation (SCA).

